# Cardiovascular care guideline implementation in community health centers in Oregon: a mixed-methods analysis of real-world barriers and challenges

**DOI:** 10.1186/s12913-017-2194-3

**Published:** 2017-04-05

**Authors:** Rachel Gold, Arwen Bunce, Stuart Cowburn, James V. Davis, Celine Hollombe, Christine A. Nelson, Jon Puro, John Muench, Christian Hill, Victoria Jaworski, MaryBeth Mercer, Colleen Howard, Nancy Perrin, Jennifer DeVoe

**Affiliations:** 1 Kaiser Permanente Northwest Center for Health Research, 3800 N. Interstate Avenue, Portland, OR 97211 USA; 2 OCHIN, Inc., 1881 SW Naito Parkway, Portland, OR 97201 USA; 3 Oregon Health Science University, 3181 S.W. Sam Jackson Park Rd., Portland, OR 97239 USA; 4 Virginia Garcia Memorial Health Center, 2935 SW Cedar Hills Blvd, Beaverton, OR 97005 USA; 5 Multnomah County Public Health Department, 426 SW Stark St, 8th Floor, Portland, OR 97204 USA

**Keywords:** Diabetes, Health services research, Physician decision support, Implementation research, Electronic health records, Qualitative research

## Abstract

**Background:**

Spreading effective, guideline-based cardioprotective care quality improvement strategies between healthcare settings could yield great benefits, particularly in under-resourced contexts. Understanding the diverse factors facilitating or impeding such guideline implementation could improve cardiovascular care quality and outcomes for vulnerable patients.

**Methods:**

We sought to identify multi-level factors affecting uptake of cardioprotective care guidelines in community health centers (CHCs), within a successful trial of cross-setting implementation of an effective intervention. Quantitative analyses used multivariable logistic regression to examine in-person patient encounters at 10 CHCs from June 2011-May 2014. At these encounters, a point-of-care alert flagged adults with diabetes who were clinically indicated for, but not currently prescribed, cardioprotective medications. The main outcome measure was the rate of relevant prescriptions issued within two days of encounters. Qualitative analyses focused on CHC providers and staff, and, guided by the constant comparative method, were used to enhance understanding of the factors that influenced this prescribing.

**Results:**

Recommended prescribing occurred at 13–16% of encounters with patients who were indicated for such prescribing. The odds of this prescribing were higher when the patient was male, had HbA1c ≥7, was previously prescribed a similar medication, gave diabetes as the chief complaint, saw a mid-level practitioner, or saw their primary care provider. The odds were lower when the patient was insured, had ≥1 clinic visits in the past year, had kidney disease, or was prescribed certain other medications. Additional factors were associated with prescribing of each medication class. Qualitative results both supported and challenged the quantitative findings, illustrating important tensions involved in guideline-based prescribing. Clinic staff stressed the importance of the provider-patient relationship in guiding prescribing decisions in the face of competing priorities and care needs, and the impact of rapidly changing guidelines.

**Conclusions:**

Diverse factors associated with guideline-concordant prescribing illuminate the complexity of delivering evidence-based care in CHCs. We present possible strategies for addressing barriers to guideline-based prescribing.

**Clinical trials registration:**

This trial was registered retrospectively.

Currently Controlled Trials NCT02299791. Retrospectively registered 10 November 2014.

**Electronic supplementary material:**

The online version of this article (doi:10.1186/s12913-017-2194-3) contains supplementary material, which is available to authorized users.

## Background

Spreading effective quality improvement (QI) strategies across healthcare settings is challenging. We studied such cross-setting translation using a diabetes care QI intervention, the ‘ALL Initiative,’ or ‘ALL.’ At Kaiser Permanente (an integrated care system serving insured patients), ALL substantially improved rates of guideline-concordant prescribing of cardioprotective statins and angiotensin-converting enzyme inhibitor (ACEI)/angiotensin II receptor blockers (ARBs) to patients with diabetes [1]. Through a cluster-randomized clinical trial (cRCT), we demonstrated the overall feasibility and effectiveness of translating ALL into community health centers (CHC) serving socioeconomically vulnerable patients [2]. We then conducted this mixed-methods assessment to better understand the multi-level factors affecting cardioprotective prescribing at CHC encounters.

In the general U.S. population, CVD-related care often does not adhere closely to care guidelines [3]. Evidence shows that less than half of individuals at risk of developing CVD are treated according to guidelines [4, 5], and rates of appropriate care are especially low in the racial/ethnic minority [4–6], uninsured [7], and low-income patient populations [8, 9] served by CHCs. A few previous studies identified factors that can impact adoption of guideline-based cardioprotective prescribing [10]. In a Veterans’ Affairs population, only 8% of clinicians intended to act on guideline-based prescribing, even when alerted to do so [11]. Their reasons included not being the patient’s primary care provider (PCP), preferring to wait until another visit, disagreement with the guidelines, or anticipated patient nonadherence. In other contexts, guideline-based ACEI/ARB prescribing was more likely among patients who were male [9] or had high blood pressure [12], and less likely if there were clinical contraindications [13]. Other factors precluding guideline-based prescribing included patient preference and clinician judgment [13]. Our objective was to contribute to this literature by providing richly described mixed methods data on the multi-level factors associated with implementation of guideline-based cardioprotective prescribing in a context where alerts were provided to notify CHC clinicians of patients indicated for such prescribing. Little such research has been conducted in this important but often neglected setting [14–17].

## Methods

We adapted Kaiser Permanente’s successful intervention, which targets guideline-based cardioprotective prescribing for patients with diabetes mellitus, through an iterative, stakeholder-driven process involving researchers, electronic health record (EHR) programmers, and CHC staff and providers, as previously described [18]. We then conducted a cluster-randomized pragmatic trial in 11 CHCs in a staggered process with six “early” CHCs implementing the intervention one year before five “late” CHCs.

CHCs serve the most vulnerable patients in the United States, whose CVD prevalence and risk are significantly higher than in the general population [19]. The study CHCs were members of OCHIN, Inc., a non-profit organization that provides health information technology, including an EpicCare© electronic health record (EHR), to CHCs [20, 21]. All OCHIN member clinics share one instance of the EpicCare EHR with a single master patient index, managed by OCHIN. The 11 study CHCs are ambulatory primary care clinics managed by three Federally Qualified Health Center systems in the Portland, Oregon metropolitan area. They vary in size and organizational structure: one is operated by a large academic medical center; six by an urban county health department; and four by a non-profit organization primarily serving suburban, Spanish-speaking populations.

The study CHCs were recruited from OCHIN’s membership, based on their willingness to participate, and their location in or near Portland, Oregon, to enable rich qualitative data collection. No sampling was involved.

Quantitative data were extracted from OCHIN’s EHR database. Verbal consent was obtained from clinic staff for qualitative data collection. The study was approved by Kaiser Permanente Northwest’s Institutional Review Board.

The quantitative analyses include all in-person encounters between June 2011 and May 2014 at the intervention CHCs, at which an ALL-related point-of care alert ‘fired,’ and the provider had prescribing privileges. Per the alerts’ criteria, these were encounters with adult (aged 18–75) patients with diabetes who were clinically indicated for a targeted medication according to national guidelines (Additional file 1: Appendix A) but did not have a prescription within the last year. (The alerts did not fire for patients with documented contraindicating diagnoses). We excluded one CHC where the alerts were unintentionally deactivated, leaving 10 CHCs in these analyses.

Our ethnographic process evaluation [22] was designed to understand the intervention and its impact from the participants’ perspective [23] to gain a rich understanding of the ‘how’ and ‘why’ behind quantitative results. Over three years, a two-person qualitative team assessed relevant care team actions and decision-making through observation of CHC workflows (126 fieldnotes), interviews and group discussions with CHC staff (42 transcripts), weekly diaries completed by clinic-based study staff (31 months), and document review (201 artifacts), from December 2011-October 2014.

### Analysis

#### Quantitative

This retrospective analysis used EHR data from the 24 months following activation of the alerts in the study CHCs. Since the study involved staggered implementation (the alerts were activated sequentially in different CHCs), calendar dates for data collection varied. The outcome of interest was whether a relevant prescription was issued within two days of patient encounters in which a point-of-care alert ‘fired.’ We examined statin prescribing rates in encounters in which a statin alert fired (‘statin encounters’), and ACEI/ARB prescribing rates in encounters in which an ACEI/ARB alert fired (‘ACEI/ARB encounters’). If both types of alert fired at one encounter, the encounter was included in both groups. Patient- and encounter-level independent variables were selected based on subject matter knowledge, previous research, data availability, and qualitative results.

We conducted descriptive analyses and checked independent variables for collinearity, choosing one for inclusion in subsequent analyses if indicated (e.g., ethnicity was highly correlated with language, so we dropped language). Since the data included repeated measures (individual patients could have multiple encounters in the data) and dichotomous outcomes, we used general estimating equations with binary outcome and a log link function [24]. We examined bivariate models for each outcome-independent variable pair, and interactions between selected variables (e.g., patient age * sex). Any variable or interaction significantly associated with the outcome at α = .10 was included in a multivariable model. Next, covariates and interaction terms were systematically removed using backward elimination (cutoff of α = .05) until a final multivariable model was specified. Only encounters with complete information on all covariates were utilized. Sensitivity analyses were performed to examine the effect of covariance structure on model fit, and a 1-dependent covariance structure was selected for the final multivariate models. As the study CHCs were operated by three service organizations, all models were adjusted by organization. Statistical analysis was performed using SAS 9.4.

#### Qualitative

Qualitative analysis was guided by the constant comparative method [25, 26], in which inductive conceptual categories (codes) are identified in a preliminary set of data; subsequent data is continually compared to those initial categories to identify commonalities and differences and confirm or challenge emerging patterns and interpretations. In this case, one year into the three-year data collection period, the two-person qualitative team undertook a process of data immersion, reflection, and discussion [27] to identify emergent categories (codes) in the data collected to date. Codes were grounded in the data, and developed prior to and independent of the quantitative results. Each of the two researchers then independently applied these codes to a sample of transcripts, compared and discussed results, and adjusted code definitions as necessary to ensure agreement; this cycle was repeated until the coders were confident that they understood the codes and had applied them consistently. The lead qualitative researcher then used QSR NVivo software to code all the qualitative data collected thus far. As additional data was collected and compared with the initial categories, code definitions were refined and additional codes added as necessary. Any disagreement or discrepancy was resolved by consensus. Identification of patterns in the data (connections between categories) and our interpretations of these patterns was informed by our time in the clinics, regular conversations among the study team, and presentation of preliminary results to clinic leadership for feedback (member checking) [28]. Qualitative coding was complete before quantitative analyses were conducted. After statistical analyses were completed, the qualitative data were used to better understand the ‘why’ behind the overall intervention outcomes.

## Results

Descriptive analyses included 11,588 statin encounters and 9,887 ACEI/ARB encounters. Only 13.2% of statin encounters and 15.8% of ACEI/ARB encounters resulted in an order for an indicated medication within 2 days (Table 1).

**Table 1:** Encounter characteristics associated with statin and ACEI/ARB prescribing at in-person office visits at 10 community health centers in Oregon where the medication(s) was/were clinically indicated^a^, June 2011 to May 2014

	Office visits^b^ where a statin indicated			Office visits^b^ where an ACEI/ARB was indicated		
	Number of encounters^c^	% with an statin Rx < = 2 days	*p*-value	Number of encounters^c^	% with an ACEI/ARBs Rx < = 2 days	*p*-value
Total	11588	(13.2)		9887	(15.8)	
Encounters						
Diabetes encounter^d^			<.0001			<.0001
# encounters for patient in last year with same provider			<.0001			<.0001
0	3114	(14.3)		2669	(21.9)	
1-3	4345	(16.8)		3455	(18.4)	
> = 4	4129	(8.7)		3763	(9.2)	
# encounters for patient in last year at same clinic			<.0001			<.0001
0	1186	(22.9)		1091	(37.2)	
1-3	4114	(18.4)		3202	(20.5)	
> = 4	6288	(8.0)		5594	(9.0)	
Diabetes encounter^d^			<.0001			<.0001
No	5082	(6.2)		4388	(8.7)	
Ye	6506	(18.7)		5499	(21.5)	
Encounter provider type			0.0003			<.0001
Physician/Resident	6810	(12.3)		6256	(13.6)	
PA/NP	4778	(14.6)		3631	(19.6)	
Encounter provider was PCP?			<.0001			<.0001
No	2092	(7.1)		1672	(10.0)	
Yes	9257	(14.5)		7988	(16.8)	
Missing/Unknown	239	(19.2)		227	(25.6)	
Nurse touched chart in encounter?			<.0001			0.0007
No	8383	(12.3)		7190	(15.1)	
Yes	3205	(15.6)		2697	(17.9)	
# non-study alerts fired at encounter			0.5615			0.0046
0	2076	(13.0)		1681	(17.3)	
1-2	6376	(13.0)		5650	(14.8)	
> = 3	3136	(13.8)		2556	(17.2)	

Abbreviations: *ACEI* angiotensin-converting enzyme inhibitor, *ARB* angiotensin II receptor blockers; *PA* Physician’s Assistant, *Pcp* primary care provider assigned to patient, *NP* Nurse Practitioner

^a^Point-of-care alert for specified drug class fired at the visit

^b^Office visits defined as in-person encounters at a study clinic where the provider was a MD, PA, NP, or resident, and the encounter Evaluation & Management CPT code was in: 99201-99205; 99212-99215; 99243; 99385-99387; 99395-99397

^c^Visit counts are not mutually exclusive, e.g., if both types of BPA fired at a visit, the visit is counted in both the statin and ACEI/ARB columns. *p*-value from chi square test for independence between characteristic and outcome of Rx < = 2 days

^d^Diabetes encounter = DM listed as chief complaint or DM listed as the primary Dx for visit, or DM was the first Dx associated with visit

**Fig. 1:**
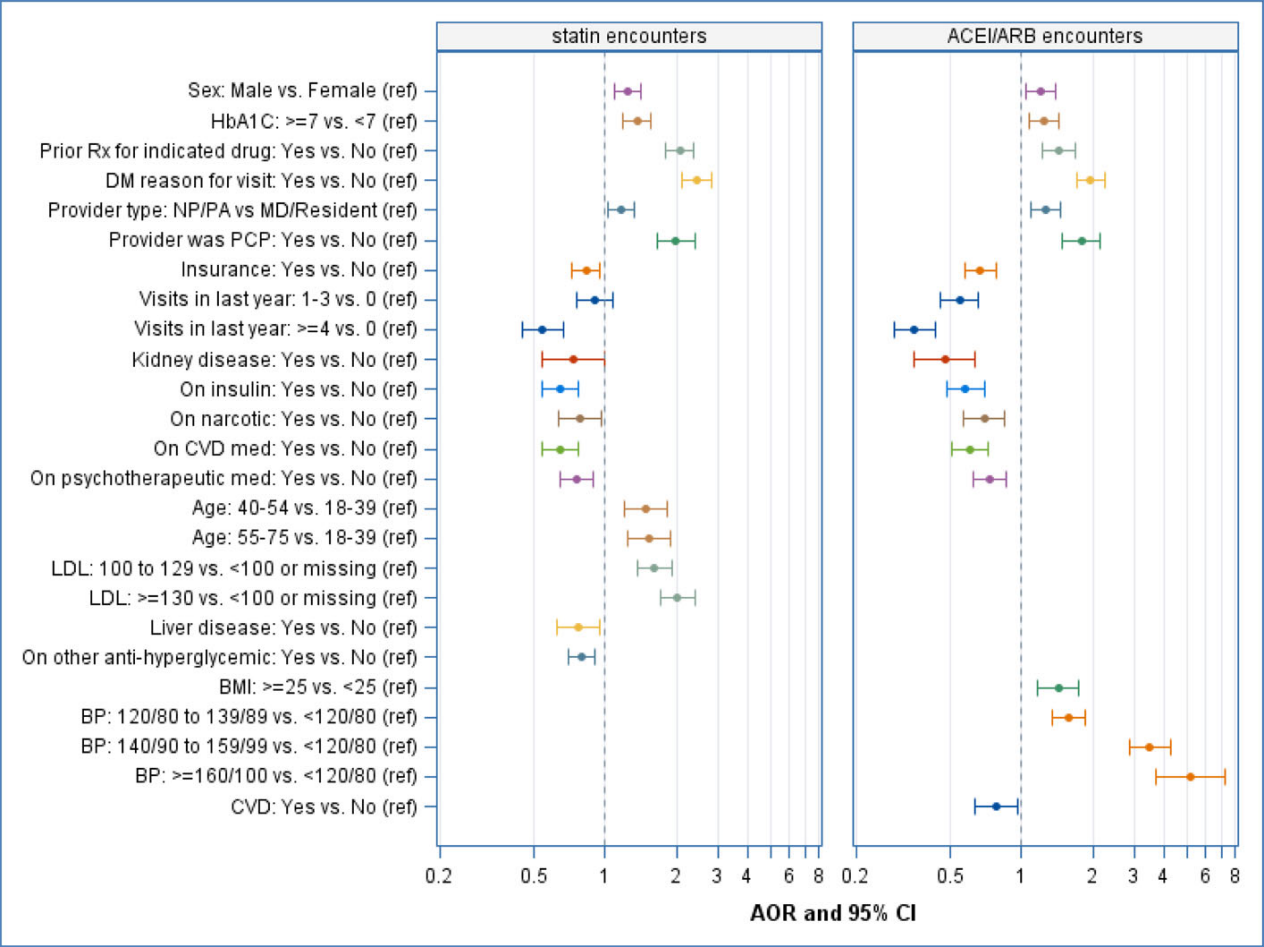
Adjusted odds ratios, 95% confidence intervals associated with statin and ACEI/ARB prescribing at in-person office encounters at 10 community health centers in Oregon, at which the medication(s) was/were clinically indicated; June 2011-May 2014. Statin encounters *n* = 11,084. ACEI/ARB encounters *n* = 9,108

In multivariable regression analyses, after excluding encounters with incomplete data, the final statin model included 11,084 encounters, and the final ACEI/ARB model included 9,108 encounters. Figure 1 shows the results of these regression analyses. For most of these regression analysis results, qualitative data provided rich explication; see Table 2.

**Table 2:** Selected excerpts from qualitative data by theme

Theme	Excerpts
Patient gender	*“And the statins, I was never really comfortable with the under forties. We have a lot of thirty-four year old women that you guys want me to put on an ACE or a statin. And I’m just not going to do it, because they're going to end up pregnant on two class X medications.”* (MD)
	*“Well, I talk to them about birth control, and if they’re on. A lot of them have had their tubes tied, so then I, you know, go ahead and prescribe it if they’re willing to take it. And then some of them are pretty unreliable with their methods, or questionable. So then I, you know, talk to them, try to get them on more reliable birth control. And then … if they’re willing to do that, then, you know, I’m willing to do it.”* (MD)
Prior prescriptions	*“I find that a fair number of my patients do have side effects to Statins and we just can’t find one that doesn’t cause them myalgias.”* (NP)
	*“I mean, it varies. But yeah, usually they [patient] don’t want to try another one [if have side effects].”* (NP)
Provider type	*“… I think historically NPs are really good at following guidelines. … Ultimately it’s going to be like, okay, well, if the … Board of Physicians has decided that this is what should be done, I’m probably going to follow it. I think there’s something about the hierarchy of education level and training.”* (NP)
	*“… I’ve practiced long enough to have known lots of MDs who are guideline resistant. You know, in terms of telling me how I have to practice kind of thing.”* (MD)
Primary care provider	*"I’m not going to manage somebody’s diabetic. If you’re a diabetic you need a home.”* (MD)
Competing needs	*“… these are fifteen minute appointments. When someone has a migraine you’re not going to be like, so enough about the migraine. Like what’s up with your insulin, you know? So I think that so many of our patients come here for acute pain needs, that this stuff is all overlooked.”* (RN)
	*“If I have a new diabetic I talk to them … [about] the fact that there are all sorts of recommendations about medications that we use to treat not only the blood sugar, but also to help prevent complications of it. And if we put you on every medicine that’s recommended you’d walk out of here and you’d have a … shopping bag full. And I say, but that’s not where we’re going to start. … I do it piecemeal. It’s so overwhelming otherwise, I think.”* (MD)
Concurrent medications	*… shared that her Russian patients do not like pills as a general rule and are highly resistant to multiple meds. In their culture, they have an old saying: "You treat one and kill the other one…. [RN] told me that this thinking is pervasive in their culture, so many patients refuse preventive meds completely … this is a real problem for her patients who … are already taking an anti-glycemic agent … will refuse any other meds because one should be enough.* (observation field note)
	*Wonders if there are any meds we can suspend or discontinue, she is feeling burdened with the amount of pill she takes … PLAN: continue ACEi … suspend statin today to reduce pill burden.* (PA encounter notes from patient chart)
Changing guidelines	*“Which is was a little unclear to me around, like if I have like a nineteen year old, you know, should I put them on a statin? And then that changed even more recently with the AHA Guidelines. And so to me it felt like a moving target.”* (Resident)
	*“[W]hat was the age cut off again? You know, it's like those things change over time. Was it forty, was it fifty? … how many other risks do they have to have before I put them on this medicine, you know?”* (RN)
Patient age	*"And you're twenty-seven years old, and I'm going to make you take a bunch of pills for the rest of your life. It's hard … I feel much better about doing it for middle-aged people with bad diabetes."* (NP)
	*“I’m not convinced that by putting someone on a tiny dose of an ACE it’s going to really reduce their cardiovascular risk if they’ve only had diabetes for two years and they’re less than fifty.”* (MD)

*Abbreviations: MD* Medical Doctor, *NP* Nurse Practitioner, *RN* Registered Nurse

### Patient gender

The odds of prescribing indicated statins and/or ACEI/ARBs were significantly *higher* in encounters where the patient was male (versus female). Qualitative data indicated common provider discomfort with giving teratogenic medications to women of childbearing age; many providers were more comfortable prescribing to women at the older end of the childbearing years. Most said they would prescribe ACEI/ARBs and statins to women of childbearing age if they felt the patient understood the risks and was on reliable birth control (variously defined). A couple of providers felt this decision should be the patient’s: “*But to not treat you for something we know you’re at huge risk for just because you might skip a pill and get pregnant … seems very paternalistic … like not really giving people the full information, the full treatment.*” Others almost never prescribed these medications to younger women*:* “*I’m totally uncomfortable with that. I’m not going to do it*.”

### Prior prescriptions

The odds of prescribing indicated statins and/or ACEI/ARBs were significantly *higher* in encounters in which the patient had a prior prescription for a medication in the indicated class (versus not). Qualitative data indicated that many patients with prior prescriptions merely needed refills. However, providers noted that patients who had side effects from a prior prescription often resisted trying another medication in that class, reporting, for example: “*They just give up and won’t take anything.*” Many reported being less likely to encourage patients with past side effects to try again, anticipating resistance, and judging that the time was better spent elsewhere.

### Type of provider

The odds of prescribing indicated statins and/or ACEI/ARBs were significantly *higher* in encounters in which the patient was seen by a mid-level practitioner (Nurse Practitioner (NP) or Physician’s Assistant (PA)), versus a physician or a resident. Mid-level providers said they may be more likely to follow care guidelines because they are “*good little soldiers*,” fear legal repercussions, have less training in analyzing evidence, and are less ‘ego-driven’ than doctors. About half of the physicians thought mid-level providers might follow guidelines better because physicians “… *feel like we should be able to practice autonomously*.”

### Primary care provider

The odds of prescribing indicated statins and/or ACEI/ARBs were significantly *higher* in encounters where the patient was seen by *their* PCP (versus another provider). Qualitative findings show that most providers said they would not start a new preventive medication for another’s patient, although some indicated they would welcome such help, and many were willing to refill another provider’s prescription. Several said they would prescribe for patients of other PCPs on their team: “*[W]hen we cover amongst our team, [we] have no problem starting somebody on Lisinopril who should be on it*.” The provider-patient relationship was important; several providers said knowledge of the patient affected when and how they prescribed the indicated medications. If they knew the patient was motivated, supported, and likely to be compliant, they were more likely to prescribe. One stated, “*I think it really depends on how well you know your patient … [and] assess your patient's ability to deal with that stuff.*”

### Competing needs

The odds of prescribing statins and ACEI/ARBs were significantly *lower* when the patient had ≥1 visits to the same clinic in the past year (versus none, or never seen at the clinic before). Qualitative data added nuance to this finding. Many providers said that they adjusted the care provided to avoid overwhelming patients at a single or first visit. One said, “*… if we load up in the beginning, a lot of times they just shut down and their adherence goes into the toilet*.” Conversely, a few said they try to prescribe all necessary medications at a single visit, but if they meet resistance, they pace introducing new medications: “*I try to do it all in the beginning … if it's not working, I try to slowly slither them in.*” Some scheduled additional visits, hoping to quickly catch the patient up on needed care. Others waited to prescribe cardioprotective medications until the patient was reliably taking blood sugar medications, and/or had controlled HbA1c; one noted: “*… some patients … it takes me years to get them on … [cardioprotective] meds*.” Patient preference (or provider perception of it) also influenced prescribing strategies; one provider noted, “… *some people just get totally overwhelmed. Other people feel like, okay, let's treat everything. Let's get started. I don’t want to die.*” The odds of prescribing indicated statins and/or ACEI/ARBs were significantly *higher* in encounters in which the patient’s chief complaint was diabetes (versus another complaint). Qualitative data showed that providers rarely have time for diabetes-related preventive care at visits where other medical needs are more acute/important to the patient. One provider said, “*People who come in a lot are often in for chronic pain. We almost never get beyond that.*”

### Concurrent medications

The odds of prescribing both statins and ACEI/ARBs were significantly *lower* when the patient had an active prescription for insulin, narcotic analgesics, CVD medications, and/or psychotherapeutics (versus not). The odds of statin prescribing were lower if the patient had a prescription for a non-insulin antihyperglycemic. As described above, providers struggled to prioritize patients’ multiple needs, and had concerns about overwhelming patients with medications. One stated: “*People who have limited resources and who are … having a hard enough time taking care of… one thing, you give them another thing, and bloop, you lose something*.” Patients sometimes refused additional medications, particularly those intended to prevent, rather than treat, illness.

### Changing guidelines

The odds of prescribing indicated statins and/or ACEI/ARBs were significantly *higher* in encounters in which the patient had HbA1c of ≥7 (versus <7), and odds of ACEI/ARB prescribing were higher for patients with blood pressure of ≥120/80 (versus <120/80). This occurred even though the intervention was based on current, evidence-based guidelines which recommended prescribing ACEI/ARBs and statins to all patients with diabetes based on age and comorbidities, regardless of blood pressure or HbA1c values – a substantial change from prior recommendations. Qualitative data showed that although most providers cautiously supported the shift from prescribing statins/ACEI/ARBs as *treatment* (based on lab values) to prescribing *preventively* against future cardiovascular disease (CVD), comfort with doing so for patients with ‘normal’ or borderline labs varied. This was particularly true for patients already on multiple medications, concurring with providers’ previously described concern about overwhelming patients. Providers and staff said it could be difficult to change from the customary focus on HbA1c to prioritize cardiovascular health; and, as above, some chose to wait to prescribe cardioprotective medications until the patient’s HbA1c was controlled. An RN noted: “*We just … autopilot to a more traditional diabetic stance, ignoring the cardiovascular aspect of it*.” Furthermore, changing guidelines bred mistrust; one provider said, “*You stay in medicine long enough and everybody wants to solve everything with X. And then, twenty years later you find out, oh, that didn’t work.*”

### Patient age

Older age (>40 versus 18-39 years) was associated with higher odds of statin prescribing. This, too, appeared to be related to changing care guidelines. Providers often noted discomfort with putting younger patients on lifelong medications with potential side effects, especially statins. One said: “*When the 20-year-old doesn’t want to go on the statin [I say] okay, just work on your blood sugar control.*” Some felt the evidence for prescribing statins to younger patients was unclear, and were unconvinced that the benefits of prescribing statins to younger people outweighed the risks; as a result, providers often did, as one noted, “*…the thing that leads to less intervention*.”

Other factors significantly associated with guideline-concordant prescribing in quantitative results (for which there was little or no qualitative data) were more clearly based on clinical factors. Statin prescribing was significantly *lower* for patients with documented liver disease, and prescribing of either medication was *lower* for patients with kidney disease. ACEI/ARB prescribing was *lower* for patients with known CVD; *higher* odds of statin prescribing were seen among patients with low density lipoprotein (LDL) ≥100 (versus <100), and *higher* odds of ACEI/ARB prescribing among those with BMI of ≥25 (versus <25).

One other factor associated with guideline-based prescribing was patient’s insurance status, for which little qualitative data existed. However, the odds of prescribing for both statins and ACEI/ARBs were significantly *lower* when the patient was insured (versus uninsured).

### Role of automated alerts

Though we could not quantify how often the EHR-based alerts were read, reactions to the alerts were consistently mentioned in the qualitative data. One common theme was that past EHR alerts (unrelated to the study) were inaccurate, creating an ingrained mistrust of all alerts. One nurse noted, “*It’s not about the tool, it’s about the culture history.*” We also heard concerns specific to the study-related alerts; providers sometimes thought these alerts were wrong when data elements used by the underlying algorithm were inaccurate in the chart (e.g., outdated prescriptions), or the algorithm specifications were not understood (e.g., prescriptions written >1 year previously were considered inactive). One provider wrote, “*I don’t want the [alert] to fire and it makes me irritable and more likely to ignore it. It needs to learn to read. I write prescriptions for 2 year [refills] because I DO want folks to keep taking their meds. Grr.”* Perceptions varied; some providers did not notice the alerts, while others said the alerts were useful reminders and improved overall awareness. One noted “*I like the [alert] because it reminds me.”* Perceptions could change; some said the alerts were effective initially, but after updating their patients’ prescriptions, they were less useful. Conversely, others grew to increasingly rely on the alerts as EHR-based alerts overall were seen as more trustworthy, as they became convinced the study alerts were appropriately tested, and/or due to mounting evidence behind the recommendations.

## Discussion

Even in the context of an intervention demonstrated to be effective through a cRCT [2], prescribing occurred at only 13–16% of encounters in which the patient was clinically indicated for an ACEI/ARB/statin (Table 2). In multivariate models, patients with diabetes were more likely to receive guideline-concordant cardioprotective prescriptions if they were male, older, or uninsured, had been previously prescribed a medication in the indicated class, were seen by an NP or PA, were seen by their own PCP, had poorly controlled A1c/BP, had few recent visits, had few other current prescriptions, or if diabetes was the chief complaint at that encounter (Tables 3, 4). Our findings generally align with previous research on factors predicting guideline-based CVD care, as outlined in the Introduction [10–13]. Like others [10], we found that guideline-based prescribing was low overall. At encounters with automated alerts, guideline-based prescribing was more likely if the patient was male [9], saw their regular provider, and had no contraindicating comorbidities [11, 13]. Prescribing was also impacted by changing care guidelines [11], and by patient preference and/or clinician judgement about what a given patient could or would take on at a given visit [11, 13]. Differing from some past research [9], we found that past utilization was associated with prescribing. Our results add to this literature by presenting mixed methods findings on multi-level factors associated with provision of guideline-based CVD care, in the CHC setting, and in the context of an overall successful QI intervention involving automated reminder alerts. We also identified that mid-level providers were more adherent to prescribing guidelines than were physicians.

**Table 3:** Patient characteristics associated with statin and ACEI/ARB prescribing at in-person office visits at 10 community health centers in Oregon where the medication(s) was/were clinically indicated^a^, June 2011 to May 2014

	Office visits^b^ where a statin indicated			Office visits^b^ where an ACEI/ARB was indicated		
	Number of encounters^c^	% with an statin Rx < = 2 days	*p*-value	Number of encounters^c^	% with an ACEI/ARBs Rx < = 2 days	*p*-value
Total	11588	(13.2)		9887	(15.8)	
Patient characteristics						
Sex			<.0001			<.0001
Male	4198	(15.7)		3819	(17.9)	
Female	7390	(11.8)		6068	(14.5)	
Age			<.0001			0.0607
18–39	1582	(12.2)		747	(17.9)	
40–54	3060	(15.5)		2473	(16.7)	
55–75	6946	(12.5)		6667	(15.3)	
Race/ethnicity			<.0001			<.0001
Hispanic	3721	(17.7)		2714	(22.2)	
Non-Hispanic White	5460	(10.8)		5035	(12.5)	
Non-Hispanic Other	2298	(11.7)		2032	(15.4)	
Missing/Unknown	109	(13.8)		106	(20.8)	
Language			<.0001			<.0001
English	6918	(10.7)		6016	(13.0)	
Spanish	3303	(18.3)		2401	(23.1)	
Other	1321	(13.6)		1435	(15.4)	
Missing/Unknown	46	(10.9)		35	(20.0)	
% of Federal Poverty Level at encounter			0.0293			0.2398
< 100%	8568	(12.9)		7294	(15.5)	
100–199%	2177	(13.1)		1888	(16.3)	
> = 200%	751	(16.8)		639	(17.8)	
Missing/Unknown	92	(12.0)		66	(21.2)	
Insurance at encounter			<.0001			<.0001
Uninsured	3817	(18.2)		2542	(26.1)	
Insured	7759	(10.8)		7330	(12.3)	
Missing/Unknown	12	-		15	-	
Patient smoking status			0.042			<.0001
Not current smoker	8917	(13.6)		7448	(16.7)	
Current smoker	2631	(11.8)		2403	(12.6)	
Missing/Unknown	40	(17.5)		36	(47.2)	
Patient has active diagnosis of CVD			0.0015			<.0001
No CVD	9984	(13.6)		7813	(17.6)	
Has CVD	1604	(10.7)		2074	(9.2)	
Patient has active diagnosis of HTN			0.1494			0.3498
No HTN	5316	(13.7)		4026	(15.4)	
Has HTN	6272	(12.8)		5861	(16.1)	
Patient has active diagnosis of liver disease			<.0001			<.0001
No liver disease	9946	(13.9)		8572	(16.5)	
Has liver disease	1642	(9.1)		1315	(11.3)	
Patient has active diagnosis of kidney disease			<.0001			<.0001
No kidney disease	10759	(13.6)		8614	(17.3)	
Has kidney disease	829	(8.1)		1273	(6.3)	
BMI			0.2842			0.0009
< 25	1266	(13.8)		1333	(12.5)	
> = 25	9914	(13.2)		8251	(16.3)	
Missing/Unknown	408	(10.8)		303	(18.8)	
BP control			0.1188			<.0001
< 120/80	3174	(12.4)		2834	(9.4)	
120/80 to 139/89	6375	(13.1)		5291	(15.2)	
140/90 to 159/99	1702	(14.7)		1459	(26.6)	
> = 160/100	334	(15.3)		301	(35.5)	
Missing/Unknown	3	(33.3)		2	-	
HbA1c control			<.0001			<.0001
< 7	5115	(10.3)		4398	(12.8)	
> = 7	6197	(15.7)		5192	(18.4)	
Missing/Unknown	276	(11.6)		297	(16.5)	
LDL control			<.0001			<.0001
< =100 or missing	4744	(9.6)		5867	(14.3)	
100 to 129	4163	(13.7)		2194	(18.0)	
> = 130	2681	(18.9)		1826	(18.1)	

Abbreviations: *ACEI* angiotensin-converting enzyme inhibitor, *ARB* angiotensin II receptor blockers, *BMI* Body Mass Index; *BP* blood pressure, *CVD* cardiovascular disease, *HbA1c* glycated hemoglobin, *HTN* hypertension, *LDL*-low-density lipoproteins, *Rx* prescription

^a^Point-of-care alert for specified drug class fired at the visit

^b^Office visits defined as in-person encounters at a study clinic where the provider was a MD, PA, NP, or resident, and the encounter Evaluation & Management CPT code was in: 99201-99205; 99212-99215; 99243; 99385-99387; 99395-99397

^c^Visit counts are not mutually exclusive, e.g., if both types of BPA fired at a visit, the visit is counted in both the statin and ACEI/ARB columns. *p*-value from chi square test for independence between characteristic and outcome of Rx < = 2 days

**Table 4:** Patient prescription history associated with statin and ACEI/ARB prescribing at in-person office visits at 10 community health centers in Oregon where the medication(s) was/were clinically indicated^a^, June 2011 to May 2014

	Office visits^b^ where a statin indicated			Office visits^b^ where an ACEI/ARB was indicated		
	Number of encounters^c^	% with an statin Rx < = 2 days	*p*-value	Number of encounters^c^	% with an ACEI/ARBs Rx < = 2 days	*p-*value
Total	11588	(13.2)		9887	(15.8)	
Patient prescription history						
Patient previously had prescription for indicated drug			<.0001			<.0001
No	6761	(10.9)		2898	(18.5)	
Yes	4827	(16.5)		6989	(14.8)	
Patient has active^d^prescription for insulin						
No	8682	(14.7)	<.0001	7075	(18.7)	<.0001
Yes	2906	(8.7)		2812	(8.7)	
Patient has active^d^prescription for non-insulin anti-hyperglycemic meds			<.0001			<.0001
No	5639	(15.5)		4914	(18.5)	
Yes	5949	(11.1)		4973	(13.2)	
Patient has active^d^prescription for narcotic			<.0001			<.0001
No	9422	(14.7)		7805	(18.1)	
Yes	2166	(6.7)		2082	(7.3)	
Patient has active^d^prescription for CVD meds			<.0001			<.0001
No	8374	(15.4)		6553	(19.5)	
Yes	3214	(7.6)		3334	(8.7)	
Patient has active^d^prescription for psychotherapeutic meds			<.0001			<.0001
No	7541	(16.1)		5996	(20.4)	
Yes	4047	(7.9)		3891	(8.8)	

Abbreviations: *ACEI* angiotensin-converting enzyme inhibitor, *ARB* angiotensin II receptor blockers, *CVD* cardiovascular disease; *Rx* referral

^a^Point-of-care alert for specified drug class fired at the visit

^b^Office visits defined as in-person encounters at a study clinic where the provider was a MD, PA, NP, or resident, and the encounter Evaluation & Management CPT code was in: 99201-99205; 99212-99215; 99243; 99385-99387; 99395-99397

^c^Visit counts are not mutually exclusive e.g. if both types of BPA fired at a visit, the visit is counted in both the statin and ACEI/ARB columns. *p*-value from chi square test for independence between characteristic and outcome of Rx < = 2 days

^d^Rx ordered < date of the visit and end date of order is null or > date of visit

The diverse factors associated with guideline-concordant cardioprotective prescribing illuminate the complexity of delivering evidence-based care, and the challenges associated with implementing up-to-date clinical guidelines, especially in CHCs. We discuss this complexity below, and present a few suggestions for how healthcare providers could address some of these barriers to guideline-concordant care. (We recognize that these approaches may not be feasible in some care settings, but present them as potential starting place for further discussion and inquiry. While it is beyond the scope of this paper to do more than make cursory recommendations, we recommend that further research be conducted on best practices for implementing the kinds of change suggested below.)

The CHC teams serving diabetic patients with multiple needs sought to avoid overwhelming patients, while giving all necessary care during the visit. This tension underlies the complexity in some of our results. Multiple needs necessitated prioritization, so CVD prevention guidelines were often secondary to other concerns, even in diabetes-focused visits. The finding that the medications were more often prescribed for patients not recently seen contradicts the qualitative results on providers’ preference for phasing in medications, and illustrates this dilemma. Patients seen more frequently often have multiple needs, pushing cardioprotective care lower on the list. Conversely, if patients come in infrequently, providers might seek to address as many needs as possible while they have the opportunity.

Since guideline-based prescribing for patients with diabetes occurs most often at diabetes-focused encounters, longer or more frequent diabetes-specific visits might enable the provision of more recommended care components. Alternative care delivery models (e.g., group visits, virtual visits, or appointments with care managers, clinical pharmacists, nurses, or mid-level staff, in addition to visits with clinicians) could facilitate patients receiving all indicated care. For patients who present with multiple complaints, additional workflow changes (e.g., follow-up phone calls to address missed needs) may also support guideline implementation. Guideline-concordant prescribing was also more likely at encounters where the patient saw their own PCP. Addressing this constraint might involve clinics ensuring that patients see their PCP, enabling real-time communication between the PCP and the provider seeing the patient, or other mechanisms to support providers prescribing to patients who are not ‘theirs.’ This result reinforces the importance of having continuity of care with a primary care provider.

Another theme involved uptake of *new* guidelines. The intervention under study was based on guidelines which substantially differed from previous guidelines, in that they recommended cardioprotective prescribing to younger patients, and moved away from prescribing based on lab values. Yet our results show that patient age and lab values were significantly associated with prescribing, perhaps because of providers’ unfamiliarity with, and/or reluctance to adopt, the new recommendations. Providers noted difficulty with moving diabetes care away from a focus on HbA1c and treatment, toward more emphasis on CVD prevention. The alerts factored in patient LDL when determining the need for a statin prescription, based on recommendations current at the time, and higher LDL was associated with statin prescribing. Nevertheless, providers reported discomfort with prescribing to patients with borderline lab results (e.g., LDL of 101, when the guidelines recommended statins if LDL >100). More recent guidelines de-emphasized LDL when prescribing statins for patients with diabetes; implementing this change may involve similar challenges. This illustrates another tension in the implementation of evidence-based care: although guidelines should be updated to reflect current knowledge, rapidly changing guidelines can create mistrust.

Addressing this issue may require strategies for presenting new evidence at the point of care, such as hyperlinks to information about why the guideline changed or relevant clinical guidelines within care alerts. Another approach might involve identifying clinic champions tasked with (and given time for) presenting new clinical guidelines to their colleagues. In some cases, providers’ hesitancy to adhere to guidelines may stem from the fact that care guidelines are based on aggregate, population-level data. Though appropriate for most patients, such data cannot speak to each patient’s needs, creating another tension as providers strive to provide care that is guideline-based and individualized/‘patient-centric.’ This might be ameliorated if guidelines were presented as guides, not dictates, with clinical judgment still considered best practice, or if related alerts included functions allowing providers to note when guidelines are inappropriate for a given patient [29, 30].

Provider ambivalence about automated alerts almost certainly helps explain why indicated prescriptions were not issued even though alerts ‘fired’ at all encounters analyzed here. Here, though the alerts were carefully vetted with various stakeholders before implementation, some post-roll-out inaccuracies were identified. For example, in some cases relevant data (e.g., external prescriptions) was entered into the EHR in a manner inaccessible to the underlying algorithm, yielding false alerts. Such inaccuracies were addressed whenever possible, but a technical solution was not always feasible. This likely contributed to provider mistrust of the alerts. Furthermore, while automated alerts can improve prescribing behaviors [31–33], they are often overridden, ‘alert fatigue’ is common, and providers often find them irrelevant [31, 34–39]. Alerts may have more impact when they target mid-level clinical staff, are more sensitive (i.e., fire in fewer cases) [36], and include explanations for the alert’s advice [29]. Another consideration here is that the alerts were just one aspect of an intervention which also included staff trainings and education, exam room posters, and in some clinics, revised care standards. Our alerts were designed to be easy to ignore, if desired; improving EHRs’ visual cues might help. Research is needed on how to maximize alerts’ impact on provider behaviors [32], e.g., which implementation strategies [40] best support uptake of new guidelines [41–46] and which are needed in combination with alerts to optimally support guideline-based care.

### Limitations

We sought to identify the multi-level factors affecting prescribing at encounters in which alerts fired, but were unable to assess whether providers actually looked at the alerts. This limits our ability to assess the alerts’ impact. Furthermore, our ability to accurately measure nurse involvement in encounters was limited, reducing our ability to assess whether nurse involvement affected outcomes.

### Conclusion

‘Translating’ effective QI strategies into CHCs serving vulnerable populations could yield great benefits, but as our results show, many factors impact CHCs’ ability to implement care guidelines. Understanding these factors is a critical step towards addressing barriers to implementing guideline-based care. This study adds to the literature on factors affecting guideline implementation by examining and bringing to light multi-level factors associated with guideline-based care in CHCs in the context of an overall successful QI intervention. Our mixed-methods approach yielded rich insights into these factors and illuminated the complexities inherent to helping CHCs stay up to date on clinical innovations. Our findings indicate the need for further research on effective methods for supporting such practice change.

## Additional file

Additional file 1: Appendix A. Publications: The Evidence Behind the ALL Initiative Medications (DOC 29 kb).

## Abbreviations

ACEI-inhibitor: Angiotensin-converting enzyme inhibitor
ALL: Aspirin, lovastatin, lisinopril
ARB: Angiotensin receptor blocker
BAA: Business associate agreement
CAD: Coronary artery disease
CHC: Community health center
cRCT: Cluster-randomized clinical trial
CVD: Cardiovascular disease
EHR: Electronic health record
KP: Kaiser Permanente
MI: Myocardial infarction
NP: Nurse practitioner
PA: Physician’s assistant
QI: Quality improvement
RE-AIM: Reach, effectiveness, adoption, implementation, maintenance
