# Supplementary material for: Cardiovascular care guideline implementation in community health centers in Oregon: a mixed-methods analysis of real-world barriers and challenges
Source: BMC Health Serv Res. 2017 Apr 5;17:253. doi: 10.1186/s12913-017-2194-3 (PMC5382420; doi:10.1186/s12913-017-2194-3)
Supplement: Additional file 1: — Appendix A. Publications: The Evidence Behind the ALL Initiative Medications (DOC 29 kb). [file 12913_2017_2194_MOESM1_ESM.doc]

**Appendix A: Publications: The Evidence Behind the ALL Initiative Medications**

- American Diabetes Association. Standards of medical care in diabetes – 2012. Diabetes Care 2012; 35, S1: S11-63.
- Antithrombotic Trialists’ Collaboration. Collaborative metaanalysis of randomised trials of antiplatelet therapy for prevention of death, myocardial infarction, and stroke in high risk patients. BMJ. 2002;324(7329):71-86.
- Baigent C, et al; CTT Collaborators. Efficacy and safety of cholesterol-lowering treatment: prospective meta- analysis of data from 90,056 participants in 14 randomised trials of statins. Lancet. 2005;366(9493):1267- 1278.
- Brenner BM, et al. Effects of losartan on renal and cardiovascular outcomes in patients with type 2 diabetes and nephropathy. NEJM. 2001;345:861-869.
- Cholesterol Treatment Trialists’ (CTT) Collaborators. Efficacy of cholesterol-lowering therapy in 18,686 people with diabetes in 14 randomised trials of statins: a meta-analysis. Lancet 2008; 371: 117–25.
- Cholesterol Treatment Trialists’ (CTT) Collaborators. Efficacy and safety of more intensive lowering of LDL cholesterol: a meta-analysis of data from 170 000 participants in 26 randomised trials. Lancet 2010; 376: 1670–81
- Collins R, et al; Heart Protection Study Collaborative Group. MRC/BHF Heart Protection Study of cholesterol- lowering with simvastatin in 5963 people with diabetes: a randomized placebo-controlled trial. Lancet. 2003;361(9374):2005-2016.
- Daly CA, et al. The effect of perindopril on cardiovascular morbidity and mortality in patients with diabetes in the EUROPA study: results from the PERSUADE substudy. European Heart Journal. 2005;26:1369-1378.
- Downs R, et al, for the AFCAPS/TexCAPS Research Group. Primary Prevention of Acute Coronary Events With Lovastatin in Men and Women With Average Cholesterol Levels: Results of AFCAPS/TexCAPS. JAMA. 1998;279(20):1615-1622.
- Fox KM; EURopean trial On reduction of cardiac events with Perindopril in stable coronary Artery disease Investigators. Efficacy of perindopril in reduction of cardiovascular events among patients with stable coronary artery disease: randomised, double-blind, placebo-controlled, multicentre trial (the EUROPA study). Lancet. 2003;362(9386):782-788.
- Fox KM, et al. Efficacy of perindopril in reducing risk of cardiac events in patients with revascularized coronary artery disease. American Heart Journal. 2007;153:629-635.
- Gaede P, et al. Multifactorial intervention and CVD in patients with type 2 diabetes. NEJM. 2003;348:383-393.
- Hayward RA, et al. Narrative review: Lack of evidence for recommended LDL Treatment Targets: A solvable problem. Ann Intern Med 2006; 145:520-530.
- HOPE. Effects of ramipril on cardiovascular and microvascular outcomes in people with diabetes mellitus: results of the HOPE study and MICRO-HOPE substudy. Lancet. 2000;355:253-259.
- HPS. Heart Protection Study of cholesterol lowering with simvastatin in 20,536 high-risk individuals: a randomised placebo-controlled trial. Lancet. 2002;360:7-22.
- HPS. The Heart Protection Study: expanding the boundaries for high-risk coronary disease prevention. Farmer JA, Gotto AM Jr. Am J Cardiol. 2003 Jul 3;92(1A):3i-9i. Review.
- Klausen K, et al. Very low levels of microalbuminuria are associated with increased risk of coronary heart disease and death independently of renal function, hypertension, and diabetes. Circulation. 2004;110:32-35.
- Law MR, et al. Quantifying effect of statins on low density lipoprotein cholesterol, ischaemic heart disease, and stroke: systematic review and meta-analysis. BMJ 2003;326:1423-.
- LIPID Study Group. Prevention of Cardiovascular Events and Death with Pravastatin in Patients with Coronary Heart Disease and a Broad Range of Initial Cholesterol Levels. NEJM 1998; 339:1349-1357
- Pedersen TR, et al. Follow-up study of patients randomized in the Scandinavian simvastatin survival study (4S) of cholesterol lowering. Am J Cardiol. 2000;86(3):257-262.
- PROGRESS Collaborative Group. Randomised trial of a perindopril-based blood-pressure-lowering regimen among 6105 individuals with previous stroke or transient ischaemic attack. Lancet 2001; 358(9287): 1033– 1041
- Pyo˘rälä K, et al. Cholesterol lowering with simvastatin improves prognosis of diabetic patients with coronary heart disease. A subgroup analysis of the Scandinavian Simvastatin Survival Study (4S). Diabetes Care. 1997;20(4):614-620.
- Sacks FM, et al. The Effect of Pravastatin on Coronary Events after Myocardial Infarction in Patients with Average Cholesterol Levels. NEJM 1996; 335:1001-1009.
- Scandinavian Simvastation Survival Study Group. Baseline serum cholesterol and treatment effect in the Scandinavian Simvastatin Survival Study (4S). Lancet 1995; 345 (8960): 1274-1275.
- Shepherd J, et al. Prevention of CHD with Pravastatin in men with hypercholesterolemia. NEJM 1995; 333:1301-1308
- Teo KK, et al. Effects of long-term treatment with angiotensin-converting-enzyme inhibitors in the presence or absence of aspirin: a systematic review. Lancet 2002; 360(9339):1037-43.
- Thompson AM, et al. Antihypertensive treatment and secondary prevention of cardiovascular disease events among persons without hypertension: A meta-analysis. JAMA. 2011;305(9):913-922.
- Wald NJ, Law MR. A strategy to reduce cardiovascular disease by more than 80%. BMJ 2003. 326:1419-.
- Weisman SM, Graham DY. Evaluation of the benefits and risks of low-dose aspirin in the secondary prevention of cardiovascular and cerebrovascular events. Arch Intern Med. 2002;162(19):2197-2202.
- Yeh RW, et al. Population trends in the incidence and outcomes of acute myocardial infarction. NEJM. 2010;362:2155-65.
- Yusuf S, et al. Effects of an angiotensin-converting-enzyme inhibitor, ramipril, on cardiovascular events in high- risk patients. The HOPE Study Investigators. NEJM. 2000;342(3):145-153.
